# Non-suicidal self-injury motivations in the light of self-harm severity indicators and psychopathology in a clinical adolescent sample

**DOI:** 10.3389/fpsyt.2022.1046576

**Published:** 2022-12-01

**Authors:** Melinda Reinhardt, Kenneth G. Rice, Zsolt Horváth

**Affiliations:** ^1^Department of Clinical Psychology and Addiction, Institute of Psychology, Eötvös Loránd University, Budapest, Hungary; ^2^Center for the Study of Stress, Trauma, and Resilience, Department of Counseling and Psychological Services, Georgia State University, Atlanta, GA, United States; ^3^Department of Personality and Health Psychology, Institute of Psychology, Eötvös Loránd University, Budapest, Hungary

**Keywords:** non-suicidal self-injury, self-harm motivations, self-harm severity, psychopathology, clinical adolescent sample

## Abstract

**Introduction:**

Adolescents with psychiatric problems are also considered a vulnerable population in terms of non-suicidal self-injury (NSSI). In the current study, we examined the associations of interpersonal and intrapersonal NSSI motivations with several NSSI severity indicators and psychopathological characteristics.

**Materials and methods:**

In a cross-sectional research design, 158 adolescents (83.5% girls; mean age = 16.10 years; *SD* = 1.49) who have received inpatient or outpatient psychiatric treatment completed the Inventory of Statements About Self-Injury, the Strengths and Difficulties Questionnaire, and the Self-Critical Rumination Scale.

**Results:**

More than two-thirds of the sample (75.3%; *n* = 119) reported at least one episode of NSSI in their life, and 45.38% (*n* = 54) have engaged in NSSI in the past month (current self-injury). The results indicated that only intrapersonal NSSI functions were linked to NSSI severity indicators (current and repetitive NSSI, versatility), interpersonal functions were not. Furthermore, a number of psychopathological features (co-occurring mental disorders, presence of a mood disorder, more internalizing mental illness symptoms, and more pronounced self-critical rumination) were associated with engaging in NSSI for intrapersonal reasons. We also identified other differences within the specific intrapersonal NSSI motivations. It should be highlighted that the anti-suicide function of NSSI behaved in exactly the opposite way as the other intrapersonal motivations.

**Discussion:**

All this points to the fact that in clinical settings, detailed assessment of NSSI motivations and severity indicators can help to develop a more effective treatment plan.

## Introduction

Non-suicidal self-injury (NSSI) as a non-adaptive coping strategy with possible serious health consequences can be based on various motivations (1), but its emotion regulation function is the most common, particularly with regard to repetitive self-harm (2). In addition, both lifetime and point prevalence of NSSI among adolescents receiving psychiatric treatment are higher than their non-clinical counterparts. Hence, a thorough investigation of NSSI functionality in clinical youth samples can contribute to a deeper understanding of the phenomenon and its relationship with various psychopathological aspects. Reflecting on this, the present study concentrated on the associations of NSSI motives, NSSI severity, and psychopathological correlates among clinical youth.

Non-suicidal self-injury includes deliberate self-harm episodes without suicidal intent that can take many different forms, such as hitting, cutting, scratching, biting, carving, or burning the body surface (3). NSSI is most common in adolescence (4) and young adulthood (5) compared to other life stages, and it has also been demonstrated in clinical settings (6). Although compared to the past, there has recently been a significant increase in NSSI in community-wide samples (4) and young adult communities (5); clinical populations already had been identified as a highly vulnerable population for NSSI. Values of around 50–70% lifetime frequency of NSSI have been consistently found in clinical adolescent populations over the last decades (7–10). However, research findings are no longer entirely consistent with respect to whether gender differences can be detected in the prevalence of NSSI. The vast majority of the studies confirm higher rates of NSSI history among females, which is much more pronounced in clinical samples (11). Based on a large clinical sample study, Victor et al. (12) reported that, compared to males, females were more likely to engage in NSSI for intrapersonal reasons, were more likely satisfy the criteria of non-suicidal self-injury disorder [NSSID; a proposed for further study identified in the DSM-5; (13)], and had higher levels of psychopathology. However, in the same study, there were no gender differences in the age of NSSI onset, the number of NSSI methods, the frequency, medical severity, and impulsivity of NSSI in the week before the survey, or the interpersonal motivation for self-harm.

Although recurrent NSSI behavior is listed as a symptom of borderline personality disorder (BPD) in the DSM-5 (13) and as the tendency to engage in self-destructive behavior in the ICD-10 (14), according to several empirical studies, non-suicidal self-injurious acts can occur across the entire spectrum of internalizing and externalizing mental disorders (15), and can occur even in the absence of mental illness (4). NSSI is often comorbid with mood, anxiety, personality, or substance use disorders (SUDs) (16), suggesting that NSSI could be a transdiagnostic factor (15). Nevertheless, it should be stressed that NSSI is most often associated with BPD (17) and eating disorders and occurs mainly with the impulsive forms of those conditions [e.g., bulimia nervosa; (18)]. Presumably, this relationship is linked to that urgency/impulsivity (19, 20) and elevated psychiatric comorbidity (21, 22) play a significant role in both disorder categories.

Impulsivity as a multifaceted construct is not only related to a wide range of psychiatric disorders but also to risky behaviors and problem behavior, like NSSI. Hamza et al.’s (23) meta-analysis pointed out that engaging in NSSI was associated with increased self-reported impulsivity, particularly a high level of negative urgency. In this context, urgency refers to the inability to resist an urge while in a negative emotional state (24). Theories explaining impulsivity [e.g., Theory of Urgency; (25)] point to similar mechanisms as theories outlining the underlying processes in NSSI. One of the central and most common functions of NSSI is immediate emotion regulation to release the person engaging in a self-harm episode from the overwhelming emotional effects of distress (26). In this context, short-term goals of emotion regulation override long-term goals, even if they have unhealthy short- and long-term consequences (27). Unhealthy remedies for aversive emotional experiences through NSSI are the basis of two major comprehensive models of self-harm. A central element of both the Experiential Avoidance Model [EAM; (28)] and the Four Function Model [FFM; (29)] is the negative urgency function of NSSI, which helps to immediately control negative emotional states derived from unmanageable affective or social experiences. People who are more impulsive are particularly vulnerable to immediate, non-adaptive management of their negative emotions via NSSI (23).

Therefore, the nature of NSSI as a transdiagnostic symptom may lie in its most crucial functions. Although it is important to note that both intrapersonal (emotion regulation-based) and interpersonal (based on social processes) motivations can underlie NSSI, the evidence so far suggests that the intrapersonal motivation basis is much more dominant (30, 31), especially in the context of recurrent/repetitive NSSI (2). Furthermore, non-adaptive emotion regulation increases the risk of NSSI regardless of gender and age (32).

Two robust factors of NSSI functionality, intrapersonal and interpersonal motivations, consistently emerge both in Klonsky and Glenn (33) and in the automatic and interpersonal factors in Nock and Prinstein (29) FFM model. In the latter model, the intrapersonal factor refers to NSSI decreasing negative and increasing rewarding emotional states (29). In the Klonsky and Glenn (33) model, the intrapersonal factor tends to blend NSSI motivation forms of coping with negative emotions. The following motives can be classified into this superior category: (1) affect regulation (NSSI serves as an act through relieving negative affective states, like anger or depression); (2) self-punishment (via NSSI, anger or disgust with the self can be expressed); (3) marking distress (through a physical wound psychological pain can be articulated); (4) anti-dissociation (refers to generating feelings instead of a sense of emptiness or unreality); and (5) anti-suicide function (avoiding suicidal thoughts or the impulse to attempt suicide). In their meta-analysis, Taylor et al. (31) highlighted the anti-dissociation function as the most prevalent NSSI motive. Other reviews emphasized that the two most common intrapersonal NSSI functions were affect regulation and self-punishment (27, 29). Self-punishment can lead to NSSI because anger and hatred toward the self can be expressed through hurting one’s body (33). These two most common NSSI functions can even be linked if NSSI reduces the negative emotions from self-criticism (34). Several studies confirmed that repetitive self-criticism can be a concomitant personality factor in NSSI (24), even after depressive symptoms were controlled (35).

The less frequent interpersonal NSSI function comprises motives that can reduce or induce specific social experiences. Taylor et al. (31) found that the most common interpersonal NSSI motivation was when someone communicated his or her distress through self-harm. However, motives such as expressing autonomy or even peer-bonding, demonstrating toughness or sensation-seeking through NSSI appeared less frequently (27).

One more fundamental factor that needs to be highlighted in relation to NSSI is its seriousness. Several severity indicators should be taken into account to get a more accurate picture of this perplexing phenomenon. Besides frequency (i.e., repetitive NSSI), engaging in multiple methods of NSSI (versatility) is likely to be linked to severe intrapersonal and interpersonal mental health problems (36). Furthermore, repetitive NSSI is primarily affected by intrapersonal NSSI motivations (2). Several researchers have highlighted the similarities between self-harm craving in repetitive NSSI and substance craving in SUDs. Nixon et al. (37) compared the experiences of adolescents engaging in repetitive NSSI with the symptoms of DSM-IV (38) Psychoactive Substance Dependence Syndrome. Results showed that 81% of the adolescents reported more than five addictive symptoms associated with repeated self-harm. This parallel was more pronounced the more frequent and severe the self-injury and inward anger. Other research has also indicated that the addictive characteristics of NSSI are associated with negative psychological states (e.g., distress) related to self-harm (39) and with co-occurring suicidal ideation (40). While some researchers [see Blasco-Fontecilla et al. (41) for a narrative review] continue to argue for the addictive nature of NSSI, others [e.g., (42)] emphasize that NSSI is better explained by emotion regulation rather than addictive processes. In a clinical adolescent sample, recurrence of NSSI was motivated by negative reinforcement (avoiding or reducing negative emotional states). In contrast, positive reinforcement (NSSI generates positive emotions) was absent (42). In addition, craving for NSSI was significantly lower compared to drug craving and was typically reported in the context of negative emotions, while craving for drugs was reported in a wide variety of emotional states. However, we should also mention that when analyzing the internet posts of people who engaged in NSSI, the addiction potential of NSSI has been clearly demonstrated (43). The most indicative SUDs criterion was urge/craving in reference to NSSI. Moreover, the user-level addictive characteristics of NSSI are closely linked to more serious versatility and more associated psychiatric disorders (43). It is also worth noting that there are attempts to treat addiction-type experiences of NSSI as a behavioral addiction. Still, that view has not been confirmed. At best, there seem to be potentially addictive aspects of NSSI, such as urge, relapse, difficulties in stopping not to do, or shame (44).

A final point worth underlining is that poor distress tolerance and deficits in emotion regulation skills are more pronounced in adolescence due to the relative immaturity of the adolescent brain in the prefrontal cortical and stressor-sensitive regions (45). Besides a similar maturational gap, a lack of experience with more controlled adult behavior may be responsible for predisposing certain teens to engage in risky behaviors impulsively (46). In addition to the age-dependent, normative emotional imbalance and the related psychosocial instability, if a psychiatric problem is also present in adolescence, risk-taking acts such as NSSI may occur. On the other hand, the maturation of neurophysiological systems during adolescence affects not only emotions but also identity (47). Against the backdrop of increased peer connections and elevated importance of social and self-acceptance, different forms of self-derogation (e.g., shame, self-devaluation, self-criticism, and self-punishment) are more likely to develop in adolescence (for a summary, see Gilbert et al. (48)). Moreover, the repetitive and excessive form of self-criticism can also be understood as a transdiagnostic factor in many mental disorders (49). In summary, difficulties managing negative emotionality, impulsivity, and forms of self-derogation are all personality factors that can be pronounced in adolescence, in several psychiatric disorders, and in NSSI.

## Aims

Although there is a relatively large body of research on NSSI in adolescents, including in clinical samples, there are few studies that explore in detail the underlying motivations of self-harm and their relationship to self-harm severity and other psychopathological phenomena. However, it is a fact that in clinical adolescent populations, the prevalence of NSSI is exceptionally high, and there is even a view that NSSI is a transdiagnostic factor in psychiatric disorders. In addition, an increase in risk behaviors has been detected in both adolescents and clinical samples. On this basis, the initial aim of this study was to examine in detail the associations of various NSSI motivations with different NSSI severity indicators and mental health indicators in an adolescent sample with a psychiatric history. An understanding of the background of getting to and engaging in self-injurious behavior and its association with psychopathological characteristics can be of considerable help in the treatment of NSSI in clinical settings. Covering the whole adolescent age spectrum in a particularly vulnerable sample, these associations were tested while controlling for age and gender.

(1) Building on previous research, it was hypothesized that specific NSSI motivations are differently related to the indices of self-harm severity and psychopathological indicators. It was assumed that the more pronounced the NSSI motivation, the more severe the self-harm itself, that is, the higher the probability of current NSSI, the more NSSI methods used (versatility) and the greater likelihood of repeating the self-injurious act (repetitive NSSI).

(2) We also hypothesized that this relationship would be stronger for intrapersonal NSSI motivations than interpersonal motivations.

(3) Finally, we assumed that a more severe mental health state (i.e., presence of comorbid psychiatric syndrome[s], more externalizing and internalizing symptoms, and higher repetitive self-criticism) is linked with higher levels of intrapersonal motivations for NSSI.

## Materials and methods

### Participants and procedure

Two hundred teenagers in five Hungarian children and adolescent psychiatric units were invited to complete a paper-based questionnaire booklet with a focus on mental health, emotion regulation, and NSSI. Three outpatient and two inpatient clinics were involved in the cross-sectional study from November 2019 to November 2021.

Exclusion criteria were defined as the inability to complete the questionnaire, for example, because of a lack of literacy skills or a severe psychiatric condition. During the 2-year period, questionnaires were administered to consecutive patients over 13 years of age who appeared in the psychiatric ward and who did not meet the exclusion criteria.

Participants completed the questionnaire booklet either at home (for outpatient clinics) or at the psychiatry clinic (inpatient participants). Before the psychologist or psychiatrist handed out the anonymous questionnaire, the patient’s ICD-10 code(s) were recorded. At the same time, no operational staff was present during the data collection events. Adolescents completed the questionnaire by themselves and without assistance.

Ultimately, 159 completed questionnaires were returned. One participant was excluded from the data analysis due to a substantially incomplete questionnaire; therefore, the analyses were carried out on a sample of 158 adolescents in psychiatric care. The mean age of this sample was 16.10 years (*SD* = 1.49), with an age range between 13 and 21 years. Most of the adolescents were girls (83.5%; *n* = 132), while 15.8% (*n* = 25) were boys and one respondent (0.6%) indicated “other” as a gender category.

Each ICD-10 code is grouped into larger categories based on the ICD-10 manual (14). The most frequent primary diagnoses were Behavioral and emotional disorders with onset usually occurring in childhood and adolescence (F90–F98; 32.9%; *n* = 48), Anxiety, dissociative, stress-related, and somatoform disorders (F40–F48; 30.1%; *n* = 44), Eating disorders (F50 in Behavioral syndromes associated with physiological disturbances and physical factors; 21.2%; *n* = 31), and Mood (affective) disorders (F30–F39; 13.0%; *n* = 19). Four adolescents (2.7%) had Pervasive and specific developmental disorders (F80–F89), and in the case of 7.6% (*n* = 12), there was not an ICD code on the questionnaire. Organic, including symptomatic, mental disorders (F00–F09), Mental and behavioral disorders due to psychoactive substance use (F10–F19), Schizophrenia, schizotypal and delusional disorders (F20–F29), and Intellectual disabilities (F70–F79) were included as secondary or tertiary diagnoses. Comorbidity occurred in 38.4% (*n* = 56) of cases.

Participation in the study was voluntary and anonymous. Written informed consent was sought from all respondents and one of their parents. The study was approved by the National Scientific and Ethical Committee (TUKEB) of Hungary’s Medical Research Council (ETT). The ETT-TUKEB is a member of the European Network of Research Ethics Committees^1^. In this context, the study was conducted in accordance with the Declaration of Helsinki (50).

Analyses were limited to adolescents (73.5% of the total sample; *n* = 119) who indicated in the NSSI measurement that they had engaged in at least one episode of NSSI in their life. Most of this subsample were girls (88.2%, *n* = 105; 10.9% were boys, *n* = 13; and one respondent [0.8%] indicated “other” for gender). The mean age was 16.09 years (*SD* = 1.58), with an age range between 13 and 21. More than half of these adolescents live in the capital (59.3%, *n* = 70), 24.6% (*n* = 29) in other cities and 16.1% (*n* = 19) in municipalities. The overwhelming majority of their parents have post-secondary (fathers: 47.0%, *n* = 54; mothers: 59.3%, *n* = 67) or secondary education (fathers: 20.9%, *n* = 24; mothers: 16.8%, *n* = 19). Nearly half of the sample have parents living together (48.7%, *n* = 57), 42.7% (*n* = 50) are divorced, and 8.5% (*n* = 10) have one parent deceased. The majority have one (31.9%, *n* = 38) or two (31.1%, *n* = 37) siblings, while 13.4% (*n* = 16) have no siblings. Around two thirds of adolescents (64.7%, *n* = 77) consider the financial situation of their family to be average, while 4.2% (*n* = 5) are very well-off, 26.9% (*n* = 32) are fairly well-off, while only 2.5% (*n* = 3) consider the socio-economic status of their family to be below average and 1.7% (*n* = 2) significantly below average. Slightly more than half (54.6%; *n* = 65) reported engaging in self-harm before the past month (previous NSSI), while 45.4% (*n* = 54) engaged in NSSI in the previous month (current NSSI). Detailed descriptions of psychopathological variables can be found in Table 1.

**TABLE 1:** Descriptive statistics of self-harm outcomes, motives, and psychopathological variables.

The number of applied self-harm methods *M* (*SD*)	5.86 (3.00)
**Previous vs. current self-harm presence *N* (%)**	
Previous self-harm Current self-harm	65 (54.60%) 54 (45.40%)
**Occasional vs. repetitive self-harm presence *N* (%)**	
Occasional self-harm Repetitive self-harm	17 (14.30%) 102 (85.70%)
**Self-harm motives**	
Affect regulation *M* (*SD*)	3.13 (1.99)
Anti-dissociation *M* (*SD*)	2.03 (1.49)
Anti-suicide *M* (*SD*)	1.55 (1.16)
Autonomy *M* (*SD*)	0.56 (0.87)
Interpersonal boundaries *M* (*SD*)	0.81 (1.07)
Interpersonal influence *M* (*SD*)	0.72 (1.09)
Marking distress *M* (*SD*)	1.34 (1.35)
Peer-bonding *M* (*SD*)	0.39 (0.90)
Revenge *M* (*SD*)	0.32 (0.83)
Self-care *M* (*SD*)	0.30 (0.75)
Self-punishment *M* (*SD*)	2.30 (1.51)
Sensation seeking *M* (*SD*)	0.60 (1.00)
Toughness *M* (*SD*)	1.28 (1.26)
Sum of interpersonal self-harm motives *M* (*SD*)	4.98 (4.13)
Sum of intrapersonal self-harm motives *M* (*SD*)	10.35 (4.04)
**Mood (affective) disorders *N* (%)**	
Absence Presence	82 (75.20%) 27 (24.80%)
Anxiety disorders* *N* (%)	
Absence Presence	74 (67.90%) 35 (32.10%)
**Disorders associated with physiological disturbances *N* (%)**	
Absence Presence	83 (76.10%) 26 (23.90%)
**Behavioral and emotional disorders with childhood and adolescence onset *N* (%)**	
Absence Presence	55 (50.50%) 54 (49.50%)
**Schizophrenia spectrum disorders *N* (%)**	
Absence Presence	108 (99.10%) 1 (0.90%)
**Intellectual and pervasive disorders *N* (%)**	
Absence Presence	106 (97.20%) 3 (2.80%)
**Co-occurrence of two or more disorders *N* (%)**	
One psychiatric disorder Two or more psychiatric disorders	68 (62.40%) 41 (37.60%)
Externalizing psychopathological symptoms *M* (*SD*)	8.85 (3.81)
Internalizing psychopathological symptoms *M* (*SD*)	10.25 (3.41)
Self-critical rumination *M* (*SD*)	29.76 (7.40)

Missing cases (*N* = 10) were not considered for computing proportions (%) on the categorical variables measuring psychiatric diagnoses. Missing cases on the variables measuring self-harm motives: *N* = 3. *The overall category of Anxiety disorders includes PTSD.

### Measures

#### Non-suicidal self-injury

Several aspects of NSSI were assessed with the Inventory of Statements about Self-Injury [ISAS; (33)]. The ISAS can be divided into two parts. The first unit comprises various descriptive and additional information about NSSI, such as lifetime frequency of 12 self-injury methods (e.g., cutting, severe scratching, hitting, biting, burning, and an additional method can be entered which was not previously included) and the estimated number of NSSI episodes for each method. The remaining questions of the ISAS are completed only by those who engaged in at least one NSSI episode at least one time in their life. The first part of the ISAS also includes questions about the first and the last engagement in NSSI, the experienced pain and being alone during NSSI, the urgency of the NSSI episode, and the desire to stop NSSI.

Three severity indicators were developed based on the questions in part one. First, versatility refers to the number of applied NSSI methods. Second, two groups can be formed based on the frequencies of different types of NSSI episodes. Those engaging in NSSI less than 10 times were categorized as “occasional self-injury,” while those reporting 10 or more lifetime episodes of NSSI were categorized as “repetitive NSSI” (51). Finally, those adolescents who engaged in NSSI before the past month (previous self-harm) were distinguished from those who engaged in NSSI in the previous month (current self-harm).

The second part of the ISAS reflects on the possible motivations or functions of NSSI (33). This study uses the short version of the second part of the ISAS (52). The 26 motivation items were rated on a 3-point scale (0 = *Not relevant*, 1 = *Somewhat relevant*, and 2 = *Very relevant*). The specific NSSI motivations compose two major factors, the intrapersonal and the interpersonal NSSI motives (33). The 16-item interpersonal functionality factor reflects the social background for self-injury (i.e., autonomy, interpersonal boundaries, interpersonal influence, peer-bonding, revenge, self-care, sensation seeking, and toughness). The 10-item intrapersonal motivation factor covers the self-regulation and emotion regulation aspects of NSSI (i.e., affect-regulation, anti-dissociation, anti-suicide, marking distress, and self-punishment). In the original long form of the second part of the ISAS, good internal consistency was detected for both interpersonal (α = 0.88) and intrapersonal factors [α = 0.80; (33)]. Reliability results based on the current study were lower but still acceptable values (α = 0.74 for interpersonal, and α = 0.70 for intrapersonal motivation scale).

#### Mental health symptoms

Externalizing and internalizing symptoms were measured by the Strengths and Difficulties Questionnaire [SDQ, (53)]. The 25-item scale can be divided into five subscales: (1) Conduct problems, (2) Hyperactivity/inattention (together form the Externalizing symptoms scale), (3) Emotional symptoms, (4) Peer relationship problems (together build up the Internalizing symptoms scale), and (5) Prosocial behavior. The first four factors collectively give a total difficulties score. Item responses are based on a scale from 0 to 2 (0 = *Not true*, 1 = *Somewhat true*, 2 = *Certainly true*). Higher scores on the symptomatic scales indicate more severe problems. In this study, we only used the Externalizing and Internalizing symptoms scales. The internal consistency of these scales in the subsample who have ever engaged in NSSI (α = 0.74 for Externalizing symptoms, and α = 0.64 for Internalizing symptoms) varied between acceptable and questionable, as well as in the original study for the total difficulties scale and subscales (53).

#### Self-critical rumination

The 10-item Self-Critical Rumination Scale [SCRS; (54)] evaluates a specific rumination type which refers to self-critical repetitive thinking and metacognitions. Items about this type of negative self-evaluation are rated on a 4-point scale (from 1 = *Not at all* to 4 = *Very much*). The one-factor structure questionnaire is supported by excellent statistical indicators, such as high internal consistency (α = 0.92 in the original study) and appropriate indexes for convergent and incremental validity (54). In the current study, the score also had high internal consistency (α = 0.88) in the subsample with a history of NSSI.

### Data analysis

#### Preliminary analyses: Bivariate correlations

As a preliminary analysis, pairwise correlations were calculated between the study variables. Correlations were evaluated between age (continuous variable), gender (dichotomous variable), the number of applied self-harm methods (continuous variable), previous vs. current self-harm presence (dichotomous variable), occasional vs. repetitive self-harm presence (dichotomous variable), the presence of specific psychopathological disorders (dichotomous variables), the presence of co-occurring two or more psychiatric disorders (dichotomous variable), specific interpersonal and intrapersonal self-harm motives (ordinal variables), overall interpersonal and intrapersonal self-harm motives (continuous variables), externalizing and internalizing psychopathological symptoms (continuous variables), and self-critical rumination (continuous variable). Correlations with the presence of schizophrenia spectrum disorders and intellectual and pervasive disorders were not calculated due to low prevalence rates. That is, Pearson correlations (i.e., between continuous variables), tetrachoric correlations (i.e., between dichotomous variables), polychoric correlations (i.e., between ordinal variables and between a dichotomous and an ordinal variable), biserial correlations (i.e., between a continuous and a dichotomous variable), and polyserial correlations (i.e., between a continuous and an ordinal variable) were calculated between the variables (55). The weighted least squares means and variances adjusted (WLSMV) estimation method was applied to calculate the bivariate correlations.

#### Multiple regression analyses

Next, multiple regression models were tested. First, the relationships between interpersonal and intrapersonal self-harm motives and outcomes of self-harm were analyzed in separate multiple regression models. Three self-harm outcomes were considered: the number of applied self-harm methods (continuous variable), previous vs. current self-harm presence (dichotomous categorical variable), and occasional vs. repetitive self-harm presence (dichotomous categorical variable). The outcome variables of these models were the before mentioned three self-harm outcomes. Multiple linear regression with the maximum likelihood robust to non-normality (MLR) method was applied to estimate the model with the outcome of the number of applied self-harm methods. However, count type dependent variables often violate assumptions of multiple linear regression (i.e., normal distribution and independence of the residuals, homoscedasticity) which can contribute to having unreliable standard errors of the regression coefficients (56). Residuals in the model showed approximate normal distribution (Supplementary Figures 1, 2) and independence (Durbin–Watson test: *d* = 2.03), and the assumption of homoscedasticity was also met (i.e., data showed random distribution in the scatterplot of the predicted values and residuals; Supplementary Figure 3). Large over-dispersion of the outcome variable was not evident based on the ratio of its variance and mean (9.01/5.86 = 1.54). Moreover, it was also suggested that with low prevalence at the minimum value of the count type outcome variable, it is an acceptable method to estimate the standard errors with the MLR estimator and treat the outcome variable as a continuous variable (i.e., six participants [5.04%] reported about the use of one self-harm method which was the minimum value on the outcome). Therefore, it was considered that the applied multiple linear regression model can be valid for the outcome of the number of applied self-harm methods. However, as a supplementary analysis, an additional model was also tested with Poisson regression (Supplementary Table 1). The two approaches yielded similar results. Thus, there were no differences in the interpretation of the predictive effects between the linear and the count model. Probit regression with the WLSMV estimation method was used for the other two models with the dichotomous categorical outcome variables (57). Interpersonal and intrapersonal motives for self-harm were simultaneously included in the models as predictor variables. The effects of age and gender were controlled in the models.

Second, two separate multiple linear regression models were specified. The outcome variables were interpersonal and intrapersonal self-harm motives. The predictor variables were the presence of co-occurring two or more psychiatric disorders, externalizing and internalizing symptoms, and self-critical rumination. The effects of age and gender were controlled in the models. The MLR estimation method was used for both models. Finally, effects of individual predictors on specific interpersonal and intrapersonal self-harm motives were examined if there were significant associations with overall interpersonal or intrapersonal motives. The outcome variables of self-harm motives were defined as ordinal variables. Therefore, probit regression with the WLSMV estimation method was used.

Two participants who engaged in NSSI before the past month and one respondent who engaged in NSSI in the previous month did not complete the second part of the ISAS; therefore they were not included in the analyses linked to NSSI motivations.

Analyses were conducted using the Mplus 8.0 statistical software (58).

## Results

### Descriptive statistics

A larger half of the adolescents (54.60%; *n* = 65) had engaged in NSSI before the past month, while a smaller proportion (45.40%; *n* = 54) have engaged in these acts within the past month. The overwhelming majority of the youth (85.70%; *n* = 102) engaged repeatedly in NSSI and mostly for intrapersonal reasons, of which affect regulation and self-punishment are the most prominent. More than one third of those who have ever engaged in NSSI have two or more psychiatric disorder diagnoses (37.60%; *n* = 41), and the most common diagnosis was within the larger category of Behavioral and emotional disorders with childhood and adolescence onset (49.50%; *n* = 54). Table 1 presents the descriptive statistics of the study variables.

### Preliminary analyses: Bivariate correlations

Table 2 presents the bivariate correlations between the study variables. Considering the aims of the present study, only significant correlations with self-harm outcomes and motives are discussed here.

**TABLE 2:** Bivariate correlations between the study variables.

	1	2	3	4	5	6	7	8	9	10	11	12	13	14	15	16	17	18	19	20	21	22	23	24	25	26	27
(1) Age	–																										
(2) Female gender (vs. male gender)	–0.21	–																									
(3) Number of applied self-harm methods	–0.10	0.25	–																								
(4) Current self-harm (vs. previous self-harm)	–0.05	0.11	0.39***	–																							
(5) Repetitive self-harm (vs. occasional self-harm)	–0.15	0.34	0.86***	0.27	–																						
(6) Mood (affective) disorders: presence (vs. absence)	0.17	–0.15	0.27*	0.29	0.30	–																					
(7) Anxiety disorders: presence (vs. absence)	0.08	0.11	–0.07	0.17	0.15	–0.30	–																				
(8) Disorders associated with physiological disturbances: presence (vs. absence)	0.08	0.50	0.21	0.18	0.14	–0.05	–0.69***	–																			
(9) Behavioral and emotional disorders with childhood and adolescence onset: presence (vs. absence)	–0.06	–0.26	0.02	–0.09	–0.16	–0.16	–0.60***	–0.64***	–																		
(10) Co-occurrence of two or more disorders (vs. one psychiatric disorder)	0.20	0.06	0.23*	0.35*	0.48**	0.73***	0.10	0.03	0.44**	–																	
(11) Affect regulation motives	–0.09	0.47**	0.33**	0.31**	0.24	0.16	0.01	–0.07	0.17	0.37**	–																
(12) Anti-dissociation motives	–0.01	0.34*	0.42***	0.28*	0.56***	0.01	0.08	–0.25	0.20	0.12	0.53***	–															
(13) Anti-suicide motives	0.09	–0.23	–0.28**	–0.14	–0.39**	0.01	–0.01	0.22	–0.15	–0.03	0.01	0.04	–														
(14) Autonomy motives	–0.33***	–0.09	–0.05	–0.06	–0.02	–0.05	–0.02	–0.09	0.28*	0.10	–0.11	–0.02	0.06	–													
(15) Interpersonal boundaries motives	–0.18	0.07	0.26*	0.02	0.39*	0.08	–0.04	0.07	0.14	0.31*	0.07	0.33**	0.03	0.26*	–												
(16) Interpersonal influence motives	0.07	0.05	–0.13	–0.10	0.02	0.16	–0.14	0.06	0.08	0.07	–0.16	–0.03	0.17	0.25*	0.09	–											
(17) Marking distress motives	–0.09	0.29	0.25**	0.25*	0.42***	0.43***	0.01	0.08	0.03	0.35**	0.33**	0.26*	0.06	0.16	0.27**	0.53***	–										
(18) Peer-bonding motives	–0.31**	–0.38*	–0.24*	–0.21	–0.36*	–0.06	–0.33*	–0.11	0.19	–0.15	–0.29*	–0.06	0.24**	0.60***	0.10	0.43***	–0.04	–									
(19) Revenge motives	–0.22	–0.07	–0.20	–0.35*	–0.07	–0.11	–0.19	–0.01	0.09	–0.11	–0.41***	–0.05	–0.01	0.21	0.22	0.30**	0.16	0.43**	–								
(20) Self-care motives	–0.14	0.13	–0.07	–0.16	0.08	0.12	–0.11	–0.30	0.24	0.11	–0.04	0.05	0.12	0.45***	0.04	0.32**	0.11	0.49***	0.02	–							
(21) Self-punishment motives	–0.06	0.17	0.47***	0.41***	0.50***	0.37**	0.14	0.16	–0.10	0.52***	0.36***	0.38***	–0.12	–0.10	0.35***	0.07	0.53***	–0.29*	0.04	–0.07	–						
(22) Sensation seeking motives	–0.08	–0.55***	0.05	–0.01	0.12	0.16	–0.04	–0.04	0.28*	0.37**	–0.10	0.08	0.00	0.37***	0.17	0.16	0.05	0.39**	0.19	0.36**	0.19	–					
(23) Toughness motives	0.06	–0.02	0.30**	0.27*	0.24	0.14	–0.01	0.14	0.10	0.10	0.05	0.26*	0.08	0.40***	0.03	0.32**	0.35***	0.25	0.08	0.40**	0.17	0.40***	–				
(24) Intrapersonal self-harm motives	–0.04	0.32*	0.40***	0.36**	0.44***	0.30**	0.09	0.04	0.05	0.41***	0.69***	0.74***	0.29***	–0.01	0.34***	0.21	0.74***	–0.15	–0.06	0.04	0.73***	0.07	0.28**	–			
(25) Interpersonal self-harm motives	–0.17*	–0.21	0.02	–0.06	0.06	0.10	–0.16	–0.02	0.26*	0.15	–0.14	0.14	0.15	0.69***	0.41***	0.58***	0.33***	0.71***	0.51***	0.62***	0.10	0.61***	0.63***	0.18*	–		
(26) Internalizing psychopathological symptoms	0.08	0.28*	0.39***	0.40***	0.42***	0.37**	0.25*	–0.17	–0.02	0.38**	0.25**	0.31***	–0.22*	–0.12	0.27**	0.09	0.33***	–0.27**	–0.16	–0.10	0.50***	0.07	0.09	0.40***	–0.01	–	
(27) Externalizing psychopathological symptoms	–0.02	–0.16	0.15	0.05	0.30*	–0.03	0.08	–0.27*	0.37**	0.29*	0.10	0.19*	–0.20*	0.17	0.10	0.04	0.06	0.06	0.26*	0.03	0.04	0.17	0.00	0.08	0.13	0.13	–
(28) Self-critical rumination	–0.03	0.26*	0.35***	0.46***	0.44***	0.37**	0.27	0.00	–0.12	0.43***	0.23*	0.32***	–0.20*	–0.13	0.32***	0.19	0.36***	–0.24*	–0.02	–0.07	0.62***	0.08	0.14	0.46***	0.06	0.56***	0.03

*N* = 118. Level of significance: *p < 0.050, **p < 0.010, ***p < 0.001. For dichotomous variables, the first category (not in parentheses) was coded with 1, while the category in parentheses was coded with 0.

#### Correlations between self-harm outcomes and motives

Significant positive and moderate correlations were shown between overall intrapersonal self-harm motives and all three outcomes of self-harm. Positive significant and moderate correlations were shown between the number of applied self-harm methods and affect regulation, anti-dissociation, self-punishment and toughness motives, whereas there were positive, significant and weak associations between the number of applied self-harm methods and marking distress and interpersonal boundaries motives. Anti-suicide and peer bonding motives had a negative significant and weak correlation with the number of applied self-harm methods.

The presence of current self-harm was positively, significantly and moderately correlated with affect regulation and self-punishment motives, in addition to positive significant and weak correlations with anti-dissociation, marking distress and toughness motives. A significant, moderate and negative correlation was shown between revenge motives and the presence of current self-harm motives.

Repetitive self-harm presence had positive, significant and strong correlations with anti-dissociation and self-punishment motives. Marking distress and interpersonal boundaries motives had positive significant and moderate correlations with the presence of repetitive self-harm. Negative significant and moderate associations were shown between repetitive self-harm and anti-suicide and peer bonding motives.

#### Correlations between self-harm motives and psychopathological variables

There was a significant, positive, and weak correlation between interpersonal self-harm motives and the presence of behavioral and emotional disorders with childhood and adolescence onset. Intrapersonal self-harm motives showed significant positive and moderate correlations with the presence of mood (affective) disorders and co-occurring two or more psychiatric disorders, internalizing symptoms, and self-critical rumination.

Affect regulation motives had positive significant and weak bivariate correlations with internalizing psychopathological symptoms and self-critical rumination, as well as positive, significant and moderate bivariate correlations with the presence of co-occurring two or more psychiatric disorders. Anti-dissociation motives presented significant, positive bivariate correlations with externalizing psychopathological symptoms (with weak strength), internalizing psychopathological symptoms and self-critical rumination (in the latter cases with moderate strength). Significant negative and weak correlations were shown between anti-suicide motives, internalizing and externalizing symptoms, and self-critical rumination. Marking distress motives correlated significantly positively and moderately with the presence of co-occurring two or more psychiatric disorders, mood (affective) disorders, internalizing psychopathological symptoms, and self-critical rumination. Self-punishment motives had significant positive and moderate correlation with the presence of mood (affective) disorders, whereas significant positive and strong bivariate correlations were presented with the presence of co-occurring two or more psychiatric disorders, internalizing psychopathological symptoms, and self-critical rumination.

Autonomy motives had a positive significant and weak correlation with the presence of behavioral and emotional disorders with childhood and adolescence onset. Interpersonal boundaries motives correlated significantly positively and moderately with the presence of co-occurring two or more psychiatric disorders and self-critical rumination, and significantly positively and weakly with internalizing symptoms. A significant negative and moderate correlation was shown between peer-bonding motives and the presence of anxiety disorders, whereas peer-bonding motives had significant, negative and weak correlations with internalizing symptoms and self-critical rumination. There was a significant positive and weak correlation between revenge motives and externalizing psychopathological symptoms. Sensation seeking motives had a significant positive and moderate correlation with the presence of co-occurring two or more psychiatric disorders, and had a significant positive and weak correlation with the presence of behavioral and emotional disorders with childhood and adolescence onset.

### Associations between self-harm motives and outcomes

Table 3 presents associations between self-harm motives and outcomes from the multiple regression analyses. Significant positive and moderate correlations were shown between intrapersonal self-harm motives and all three outcomes of self-harm – when the effects of age, gender, and interpersonal motives were controlled.

**TABLE 3:** Standardized regression coefficients on the associations between self-harm motives and outcomes.

	Outcome variables		
	Number of applied self-harm methods β (*SE*)	Current self-harm (vs. previous self-harm) β (*SE*)	Repetitive self-harm (vs. occasional self-harm) β (*SE*)
Age	–0.08 (0.09)	–0.05 (0.12)	–0.12 (0.11)
Female gender (vs. male gender)	0.05 (0.09)	–0.07 (0.12)	0.14 (0.12)
Interpersonal motives	–0.06 (0.10)	–0.15 (0.12)	–0.01 (0.13)
Intrapersonal motives	0.40 (0.08)***	0.40 (0.11)***	0.41 (0.15)**
Explained variance (*R*^2^)	17%	15%	23%

*N* = 115. β (SE), standardized regression coefficients with standard errors. Level of significance: **p < 0.010; ***p < 0.001.

### Associations between self-harm motives and psychopathological variables

Findings of the multiple regression analyses regarding the relationships between self-harm motives and psychopathological variables are presented in Table 4. In the multiple regression model, age was significantly and negatively associated with interpersonal self-harm motives. Moreover, co-occurring two or more psychiatric disorders and higher levels of self-critical rumination were significantly and positively associated with elevated rates of intrapersonal self-harm motives.

**TABLE 4:** Standardized regression coefficients on the associations between psychopathological variables and self-harm motives.

	Outcome variables	
	Interpersonal motives for self-harm β (*SE*)	Intrapersonal motives for self-harm β (*SE*)
Age	–0.24 (0.10)*	–0.04 (0.08)
Gender^1^	−0.16 (0.11)	0.12 (0.08)
Co-occurring two or more psychiatric disorders^2^	0.16 (0.10)	0.18 (0.08)*
Externalizing symptoms	0.06 (0.09)	–0.04 (0.07)
Internalizing symptoms	−0.09 (0.12)	0.18 (0.10)
Self-critical rumination	0.04 (0.11)	0.35 (0.10)***
Explained variance (*R*^2^)	10%	35%

*N* = 105. β (*SE*), standardized regression coefficients with standard errors. Level of significance: *p < 0.050; **p < 0.010; ***p < 0.001. ^1^Coded as: 0 = Males, 1 = Females. ^2^Coded as: 0 = Presence of one psychiatric disorder, 1 = Presence of co-occurring two or more disorders.

Due to the significant relationships between overall intrapersonal self-harm motives and psychopathological variables, multiple regression estimates were calculated between specific intrapersonal self-harm motives (affect regulation, anti-dissociation, anti-suicide, marking distress, self-punishment motives) and psychopathological variables. These results are summarized in Table 5. Higher rates of self-critical rumination were significantly associated with elevated levels of anti-dissociation and self-punishment motives. Furthermore, a significant and positive link was demonstrated between the presence of co-occurring two or more psychiatric disorders and self-punishment motives, in addition to a significant and negative effect of externalizing psychopathological symptoms on anti-suicide motives. Female gender was associated with increased rates of affect regulation motives. None of the predictor variables had significant relationships with marking distress motives in the regression model.

**TABLE 5:** Standardized regression coefficients on the associations between psychopathological variables and specific intrapersonal self-harm motives.

	Outcome variables				
	Affect regulation motive β (*SE*)	Anti-dissociation motive β (*SE*)	Anti-suicide motive β (*SE*)	Marking distress motive β (*SE*)	Self-punishment motive β (*SE*)
Age	–0.09 (0.11)	0.04 (0.10)	0.05 (0.10)	–0.16 (0.10)	–0.05 (0.09)
Gender^1^	0.24 (0.10)*	0.19 (0.12)	−0.13 (0.09)	0.05 (0.12)	–0.01 (0.11)
Co-occurring two or more psychiatric disorders^2^	0.22 (0.13)	−0.08 (0.11)	0.08 (0.12)	0.19 (0.12)	0.23 (0.09)*
Externalizing psychopathological symptoms	–0.02 (0.11)	0.16 (0.11)	−0.23 (0.10)*	–0.04 (0.11)	–0.09 (0.09)
Internalizing psychopathological symptoms	0.13 (0.14)	0.13 (0.13)	−0.09 (0.13)	0.19 (0.13)	0.18 (0.10)
Self-critical rumination	0.12 (0.14)	0.29 (0.12)*	−0.10 (0.11)	0.23 (0.14)	0.52 (0.08)***
Explained variance (*R*^2^)	22%	22%	11%	25%	55%

*N* = 105. β (*SE*), standardized regression coefficients with standard errors. Level of significance: *p < 0.050; **p < 0.010; ***p < 0.001. ^1^Coded as: 0 = Males, 1 = Females. ^2^Coded as: 0 = Presence of one psychiatric disorder, 1 = Presence of co-occurring two or more psychiatric disorders.

## Discussion

Despite a decades-long trend of a persistently high prevalence of NSSI in clinical adolescent samples, there are not sufficient enough studies examining different aspects of NSSI in depth among youth with mental illness. This study, therefore, focused on examining the motivational basis and severity of NSSI in light of several psychopathological characteristics.

The present study also confirmed the high prevalence of NSSI detected in clinical adolescent samples with a lifetime prevalence of 75% and point prevalence (i.e., current self-harm) of 45% [cf., (8)], indicating the particular vulnerability of this population in this respect as well. Moreover, the vast majority (more than 85%) of adolescents in psychiatric care reported repetitive [≥10 episodes of; (51)] NSSI in their lifetime. Another severity factor, the number of applied NSSI methods (an average of 6), was also similarly high as in previous clinical population-based studies [cf., (10, 59)]. Engaging in NSSI for intrapersonal reasons was twice as common as for interpersonal reasons. Also similar to the results of previous studies [cf., (27, 29, 31)], the most prominent motives for NSSI were affect-regulation, self-punishment, and anti-dissociation; the least frequent were such interpersonal motivations as self-care and revenge to someone via NSSI. In a clinical context, more serious self-injury seems to be more of a behavioral act regulating internal states and less of a socio-environmental relationship regulation mechanism.

Even after controlling for age, gender, and interpersonal motivational effects, intrapersonal motivation was associated with the three severity indicators of NSSI. The more dominant intrapersonal motivations lie behind self-injurious behavior, the more current, repetitive, and diversified NSSI could be expected. For all three indicators of self-harm severity, the proportion of explained variance is relatively high. For example, engaging in self-harm to regulate internal psychological states explains by itself almost a quarter of the repetitive NSSI variance. It should also be emphasized that age and gender were not associated with any of the NSSI severity indicators. These results support the line of research in which disturbed emotion regulation is closely tied with the risk of more NSSI, regardless of age and gender (32).

Intrapersonal motives behind NSSI can be interpreted as an attempt to regulate unwanted psychological states. But it can easily become a vicious circle, which may establish that behavioral addictive characteristics develop in relation to NSSI [c.f., (37)]. As the Experiential Avoidance Model records, NSSI can temporarily reduce the intensity of overwhelming emotions, and thus provides temporary relief by generating physical pain (28). However, this process, through negative reinforcement, generates the recurrence of NSSI, and becomes an automatic coping strategy for the individual. This process is in line with research findings which pointed out that higher emotional instability, anxiety, and irritability were strongly associated with poorer functioning of inhibitory processes, particularly with impulsivity and hostility (60). On this basis, it can be reasoned that the tendency to experience frequent negative and unstable emotions can significantly impair the ability to inhibit stopping someone’s motivation for engaging in NSSI. Further longitudinal studies can shed light on what influences may be involved in the association of repetitive self-harm with addictive characteristics (41).

A closer look at the intrapersonal NSSI motives, anti-dissociation, self-punishment, and marking distress are robustly associated with current and repetitive NSSI, as well as with NSSI versatility. However, affect regulation was only linked to current NSSI with diverse methods. This finding may indicate that emotion regulation could underlie both the trial of as well as repeated NSSI. However, repetitive NSSI is sustained by more specific intrapersonal motivations such as escape from an unwanted emotional condition or cognitive state, managing excessive distress, or high degree of self-deprecation.

Several studies have identified repetitive NSSI as a significant risk factor for suicidal intentions and behavior (61). Based on a literature review, repetitive NSSI was described as a precursor of suicidal behavior in the presence of a co-occurring psychiatric disorder (62). Moreover, Kiekens et al. (63) also demonstrated that NSSI is a risk factor for subsequent suicidal ideation, plans, and experiments when the presence of mental disorders was controlled. Klonsky et al. (64) pointed out that suicide attempts were predicted by repetitive NSSI and suicidal ideation, after controlling for depression, anxiety, or impulsivity. They explained the prominent role of NSSI in suicide risk by the fact that repetitive self-injury increases a person’s self-inflicted pain and aggression and thus the motivation to commit suicide. Particularly interesting in this perspective are our findings that the anti-suicidal (inhibiting suicidal ideation) function of NSSI was associated with lower severity indicators of self-harm and lower externalizing and internalizing symptomatology. The more the self-harm is motivated by the need to get rid of suicidal thoughts or urges, the less likely the person is to engage in self-harm repeatedly and in multiple ways. This may support the idea that during the initial phase of engaging in NSSI, self-harm is experienced as a barrier for suicidal ideation. Later, however, when NSSI becomes more severe, repetitive, and more extensive in its methods, it can decrease the threshold of self-inflicted pain and aggression (64). Progressing in time, this can be combined with an increase in mental health symptoms. This idea also fits into the spectrum approach to non-suicidal and suicidal behavior (65) or such complex models as the Gateway Theory (66) or Joiner’s Theory of Acquired Capability for Suicide (67). In the latter model, NSSI can serve as a habituation phase before suicide, during which people become accustomed to painful and provocative experiences that can lead to irreversible self-harm. It is also hypothesized that the relationship between NSSI and suicidal self-injury (SSI) is moderated by the severity of NSSI. That is, a stronger association between NSSI and SSI is supposed among those with more severe form(s) of NSSI (66). In summary, it is worth noting that the anti-suicide function of NSSI behaves in a unique way, different from other intrapersonal self-injury motives, and therefore deserves special attention in terms of suicide risk evaluation.

Co-occurring mental disorders, mood disorders, internalizing symptomatology, and self-critical rumination were associated with intrapersonal motivation. Co-occurring mental disorders and self-critical rumination explained substantial variation (35%) in intrapersonal functionality of NSSI. Looking at each NSSI intrapersonal motives separately and in relation to psychopathological factors gives a more nuanced picture. Internalizing symptoms were associated with all NSSI intrapersonal motivations. The more emotional problems, the more likely NSSI is motivated by emotion regulation, anti-dissociation, marking distress, and self-punishment functions, but less likely to be motivated by anti-suicide functions. Numerous studies have confirmed that high levels of depressive and anxiety symptoms are major risk factors for NSSI (68), which can be an ineffective attempt to reduce negative affective states.

Elevated self-critical rumination nearly showed a similar picture. Higher repetitive self-criticism was associated with raised affect regulation, anti-dissociation, and self-punishment NSSI functions, but a lower probability of anti-suicide function. Co-occurring mental disorders linked to affect regulation, marking distress, and self-punishment NSSI functions. The presence of a mood disorder was related to NSSI against a background of marking distress and self-punishment. Female gender was associated with more explicit affect regulation and anti-dissociation NSSI functions. This effect may be because difficulties with emotion regulation (e.g., rumination) and mood (e.g., depressive) symptoms identified as risk factors for NSSI (69) are more common among females (70).

A more rigorous analysis, that controls for additional variables when analyzing the relationship between two phenomena, showed that self-critical rumination had the strongest effect. The higher the self-critical rumination, the more likely NSSI is to be used for anti-dissociation and self-punishment. Repetitive self-critical self-focus appears to be a key factor in self-injurious behavior in adolescents with mental illness. Elevated perseverative negative self-evaluation is pervasively linked to self-punishment and a desire to escape from an emotionless or numb state through self-harm. According to the integrated model of NSSI (26), elevated self-criticism, especially if it permeates personality and thinking, is a special vulnerability factor for NSSI as engaging in self-injury is a form of self-punitive aggression. A consistent body of research suggests that those who engage in repetitive NSSI report higher levels of such cognitive distortions like self-deprecation, pervasive self-criticism, and low self-esteem (35). NSSI can be an unhealthy coping strategy to decrease distress derived from pervasive self-related pessimistic and criticizing cognitions (71). On the other hand, a significant proportion of people who engaging in NSSI claim to engage in self-harm to “punish themselves” or to “express anger toward themselves” (27). Self-deprecation may be the variable that leads some people to self-injure. Indeed, research suggests that people who have strong negative emotionality but average or low self-depreciation when upset are more likely to blame others or become aggressive toward others. Conversely, those with expressed negative emotions associated with strong self-deprecation are more likely to involve in NSSI. This suggests that high emotional dysregulation and high self-deprecation are the greatest risk for recurrent NSSI (1). Moreover, we may also assume that increased repetitive self-criticism with its intrusiveness can lead to self-depreciation and self-blaming. Therefore, an unwanted emotional state can result in dissociative symptoms (coherent functioning of identity is impeded) and NSSI can temporarily interrupt these dissociative processes by causing physical pain. At this point, we should highlight the research-confirmed links that childhood maltreatment reinforces both self-blame and self-related pessimistic attributional style (72) and predisposition to dissociation (73), as well as the later occurrence of repetitive NSSI (74). Although, childhood maltreatment was not measured in this research, it raises the possibility that those in our sample who reported more severe NSSI, particularly engaging self-harm to terminate their dissociative states or punish themselves through physical pain, may be more likely to be subjected to various forms of childhood maltreatment. These possible multiple associations should be tested in further research.

### Clinical implications

Given the persistently high rates of NSSI in clinical adolescent populations, it remains important to embed research findings in clinical practice. Our results emphasize the value of including screening questions on NSSI alongside questions on the suicide spectrum at adolescent psychiatric admission. At the next stage, it is particularly important to assess multiple indicators and motivations of self-harm in those involved in NSSI. On this basis, groups based on NSSI severity can be identified at a triage stage. This could be followed by an immediate, targeted group intervention for the most vulnerable group (with repetitive, multicausal, intrapersonal motivational background NSSI). This targeted intervention can be particularly important in adolescence, one of the most vulnerable periods of life to change. Treatment activities that strengthen healthy emotion regulation mechanisms could reduce negative, self-critical self-evaluation and inflexible attitudes and behaviors. At the same time, facilitating meaningful activities that match the adolescent’s interests and help to elaborate on unanchored emotions and negative affective states can also be useful. There is evidence that people who engage in NSSI experience fewer positive emotions (75), therefore, in addition to addressing negative emotional states, it is also important to help to find those states and activities that are rewarding instead of self-injury, and to experience more effectively the positive feelings that emerge when doing so. Correction of emotion regulation deficits can curb the negative psychological and physical consequences of self-injury, thereby promoting greater personality integration and sustained, constructive adolescent development. In parallel fashion, correcting emotional regulation deficits may even decrease the symptom severity of the associated psychiatric problem.

### Limitations

In addition to its novelties, the present study has several limitations. (1) First, the modest sample size should be considered. Although it is a difficult population to reach, the small sample made it impossible to carry out more complex statistical analyses. (2) Furthermore, we were not able to collect information on those to whom we gave the questionnaire, but for some reason, they did not return it. Since the participation in the survey was voluntary, and the purpose of the research was known, it must be taken into account that some adolescents may have filled the questionnaire because of their involvement or may not have returned it because of the sensitive topic. (3) The cross-sectional research design failed to investigate causality and follow up developmental pathways in NSSI aspects and these correlates. (4) Moreover, the inbalanced gender ratio could also have affected the results. However, the very low proportion of boys in the current study is consistent with previous studies in similar populations. To counteract this effect, effects of gender were controlled in all analyses. (5) Furthermore, the analyses did not control for the impact of the different stages of the COVID-19 pandemic. Although the time of questionnaire completion was recorded, as the COVID-19 outbreak occurred while the survey was in progress, we were unable to include questions on the psychological impact of the pandemic. (6) Although we asked the adolescents to fill in the questionnaire on their own, we were unable to check that they had done as instructed. In this context, neither symptom simulation nor dissimulation could be controlled. However, the anonymous and self-administered questionnaire also allowed us to capture more honest and valid responses that show the adolescent experience. (7) Finally, the internal consistency of the SDQ subscale of internalizing psychopathological symptoms was relatively low which should be considered when interpreting the findings with self-harm motives and outcomes.

## Conclusion

Rates of intentional but not suicidal self-harm acts are noticeably high in clinical adolescent populations. However, detailed characteristics and the role of NSSI functions is under-analyzed in these multi-vulnerable groups. The present study, therefore, focused on the relationship of NSSI motivation and severity indicators as well as psychopathology. Only intrapersonal motivations were associated with NSSI severity indicators and the latter of which can be linked to behavioral addictions. The stronger the presence of intrapersonal motivation to self-harm, the more likely that NSSI episodes are more frequent and more likely to occur by more than one method. As this is also linked to an increased risk of suicidal spectrum, it is essential to assess the motivational basis and severity indicators of NSSI in clinical settings. The accumulation of NSSI episodes and methods is closely related to deficits in emotion regulation. Thus, NSSI severity can be seen as a prolonged behavioral manifestation of emotion dysregulation (24). This speculation is reinforced by the connectedness of co-occurring psychiatric disorders along with increased internalizing symptoms and self-critical rumination to intrapersonal NSSI functionality. Elaborated scanning of certain NSSI characteristics can contribute to developing and strengthening healthy emotion regulation processes, thereby preventing mental illness transitions from adolescence into adulthood.

## Data availability statement

The raw data supporting the conclusions of this article will be made available by the authors, without undue reservation.

## Ethics statement

The study, involving human participants, was reviewed and ethically approved by the National Scientific and Ethical Committee (TUKEB) of Hungary’s Medical Research Council (ETT) (Reference number: 30905-5/2019/EKU; date of issue: 13 September 2019). The ETT-TUKEB is a member of the European Network of Research Ethics Committees (http://www.eurecnet.org/information/hungary.html). The work was conducted in accordance with the Declaration of Helsinki. Participation in the study was voluntary and anonymous. Written informed consent to participate in this study was provided by all of the respondents and one of their parents/legal guardians.

## Author contributions

MR conceived of the study, participated in its design, data collection, data analysis and interpretation of the data, and drafted the manuscript. KR participated in the interpretation of the data and helped to draft the manuscript. ZH performed the statistical analyses and helped to draft the manuscript. All the authors approved the final manuscript for submission.

## Footnotes

1
http://www.eurecnet.org/information/hungary.html
