# Supplementary material for: Non-suicidal self-injury motivations in the light of self-harm severity indicators and psychopathology in a clinical adolescent sample
Source: Front Psychiatry. 2022 Dec 1;13:1046576. doi: 10.3389/fpsyt.2022.1046576 (PMC9751932; doi:10.3389/fpsyt.2022.1046576)
Supplement: Supplementary file 1 [file Data_Sheet_1.PDF]

Supplementary Table 1. Poisson regression model showing the predictive effects on the number of applied self-harm methods

| | Outcome variable:<br>Number of applied self-harm methods<br>B (S.E.) |
|---|---|
| Age | -0.03 (0.03) |
| Female gender (vs. male gender) | 0.10 (0.19) |
| Interpersonal motives | -0.01 (0.01) |
| Intrapersonal motives | 0.05 (0.01)*** |

Notes. B (S.E.): unstandardized regression coefficients (with the corresponding standard error). Level of significance: ***p<0.001.

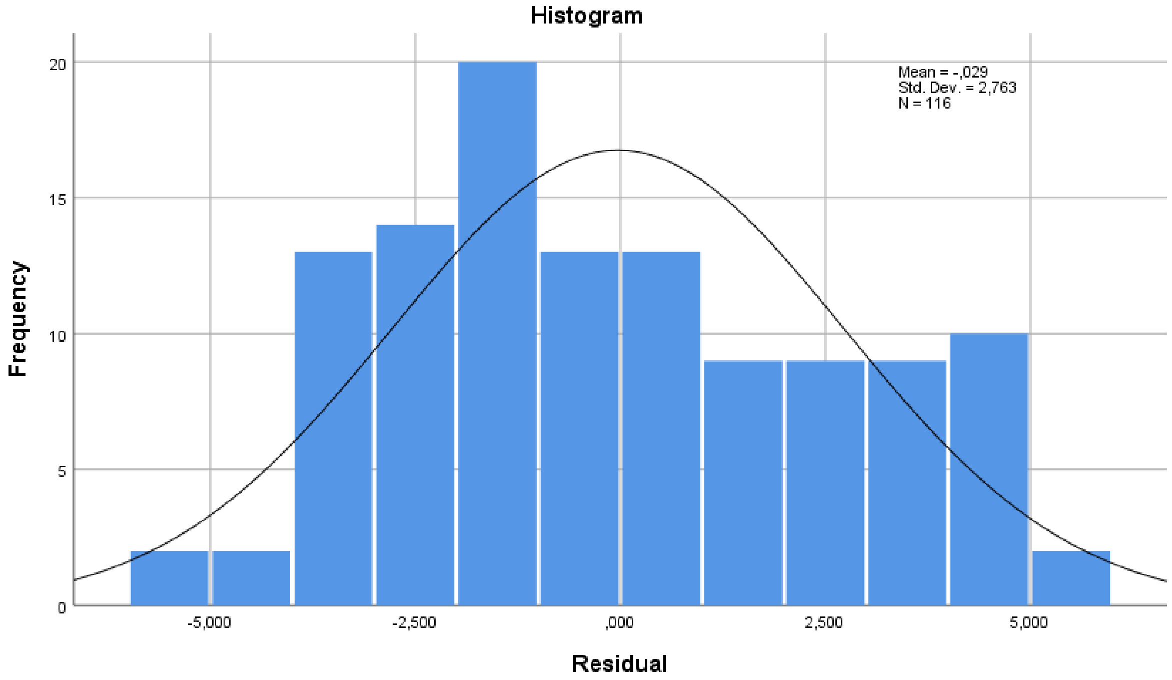


Supplementary Figure 1. Distribution of the residuals.

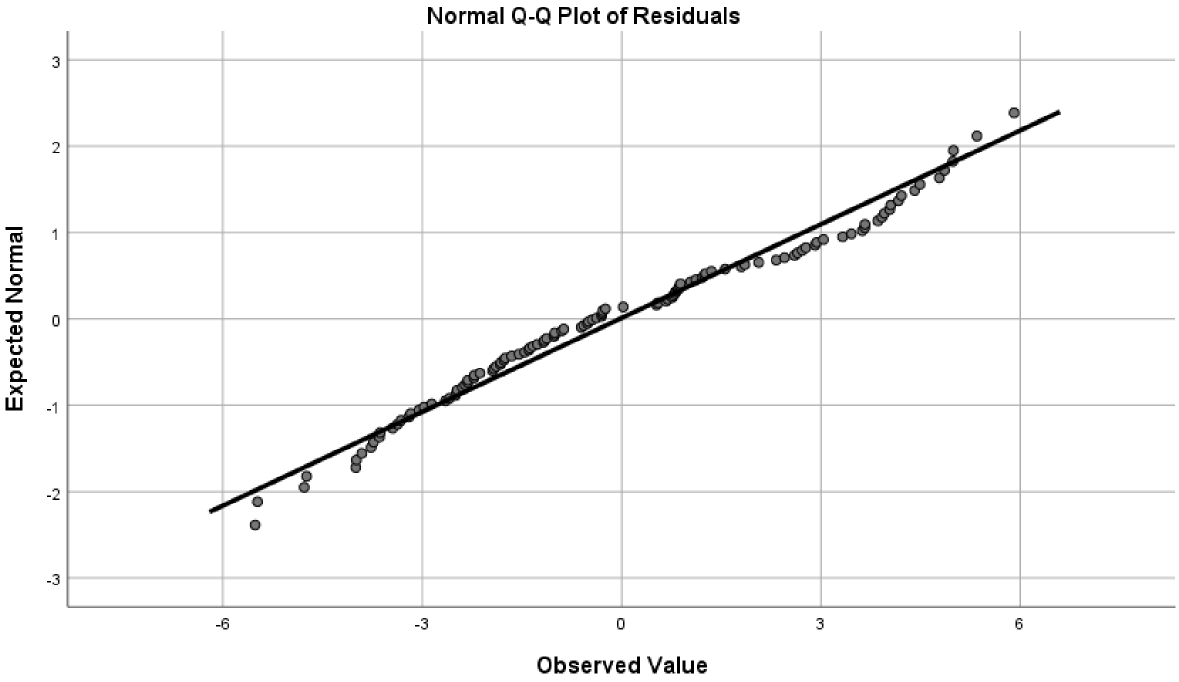


Supplementary Figure 2. QQ-plot of the residuals.

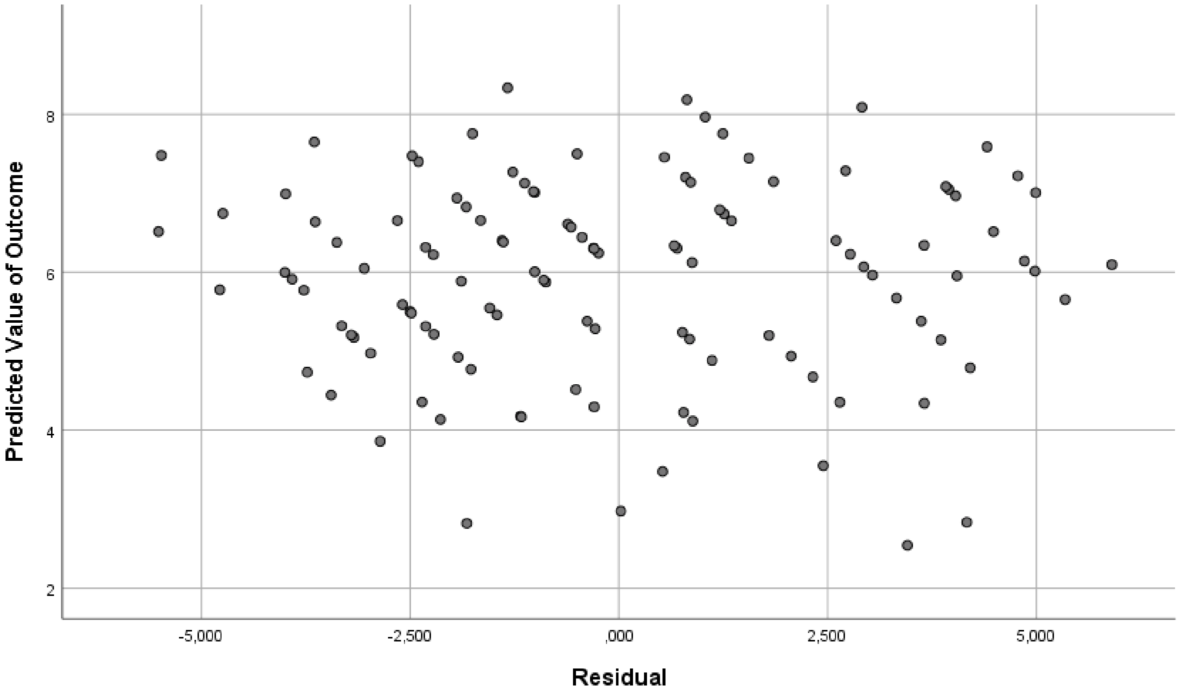


Supplementary Figure 3. A scatterplot of the residuals and the predicted outcome values for testing the assumption of homoscedasticity.
